# Gender effect on neurodegeneration and myelin markers in an animal model for multiple sclerosis

**DOI:** 10.1186/1471-2202-13-12

**Published:** 2012-01-24

**Authors:** Alessandro Massella, Giulia D'Intino, Mercedes Fernández, Sandra Sivilia, Luca Lorenzini, Silvia Giatti, Roberto C Melcangi, Laura Calzà, Luciana Giardino

**Affiliations:** 1 Health Sciences and Technology - Interdepartmental Center for Industrial Research (HST-ICIR), University of Bologna, Via Tolara di Sopra 50, 40064 Ozzano Emilia, Italy; 2 Department of Veterinary Medicine, University of Bologna, Via Tolara di Sopra 50, 40064 Ozzano Emilia, Italy; 3 Dept. of Endocrinology, Pathophysiology and Applied Biology - Center of Excellence on Neurodegenerative Diseases, University of Milan, Via Balzaretti 9, 20133 Milano, Italy

**Keywords:** experimental allergic encephalomyelitis, gender-related, rat, spinal cord, cerebellum, neurotrophins and related receptors

## Abstract

**Background:**

Multiple sclerosis (MS) varies considerably in its incidence and progression in females and males. In spite of clinical evidence, relatively few studies have explored molecular mechanisms possibly involved in gender-related differences. The present study describes possible cellular- and molecular-involved markers which are differentially regulated in male and female rats and result in gender-dependent EAE evolution and progression. Attention was focused on markers of myelination (MBP and PDGFαR) and neuronal distress and/or damage (GABA synthesis enzymes, GAD65 and GAD67, NGF, BDNF and related receptors), in two CNS areas, i.e. spinal cord and cerebellum, which are respectively severely and mildly affected by inflammation and demyelination. Tissues were sampled during acute, relapse/remission and chronic phases and results were analysed by two-way ANOVA.

**Results:**

1. A strong gender-dependent difference in myelin (MBP) and myelin precursor (PDGFαR) marker mRNA expression levels is observed in control animals in the spinal cord, but not in the cerebellum. This is the only gender-dependent difference in the expression level of the indicated markers in healthy animals; 2. both PDGFαR and MBP mRNAs in the spinal cord and MBP in the cerebellum are down-regulated during EAE in gender-dependent manner; 3. in the cerebellum, the expression profile of neuron-associated markers (GAD65, GAD67) is characterized by a substantial down-regulation during the inflammatory phase of the disease, which does not differ between male and female rats (two-way ANOVA); 4. there is an up-regulation of NGF, trkA and p75 mRNA expression in the early phases of the disease (14 and 21 days post-immunization), which is not different between male and female.

**Conclusions:**

It is reported herein that the regulation of markers involved in demyelination and neuroprotection processes occurring during EAE, a well-established MS animal model, is gender- and time-dependent. These findings might contribute to gender- and phase disease-based therapy strategies.

## Background

Multiple sclerosis (MS) is an inflammatory demyelinating disease of the central nervous system (CNS), which can progress over decades. The progressive failure of remyelination leads to the cumulative loss of axons, grey matter atrophy and prevalent neurodegeneration responsible for chronic disability and cognitive decline [1]. There is a considerable difference in the way MS affects females and males, as has been highlighted by epidemiology studies and MRI analyses [2-4]. The way of gender-influence in MS is complex and still obscure. From a pathogenic point of view, females tend to have stronger Th1-mediated immune responses and are more prone to develop autoimmune diseases, including MS [5]. However, gender might influence white matter establishment and maintenance of the mature structure of white tracts, thus affecting their repair capability. A review of in vivo imaging studies suggests that gender-related differences in white matter in the human brain exist in healthy subjects [6] and affect in particular age-related changes in precentral, cingulate, and anterior temporal white matter areas [7]. As well as in the normal brain, white matter pathology seems also to differ in males and females in several neurological and psychiatric conditions. For example, in schizophrenia there is a subtle and gender-dependent alteration in the forebrain commissures, and the neurotoxic effect of metamphetamine on frontal white matter seems to be less prominent in women than in men [8]. Sex differences in lesion size, neuronal cell loss, and mortality rates have been observed after ischemia and trauma [9]. Moreover, a gender effect in the progression of several neurodegenerative diseases has been noted. Epidemiological data point to women's proneness to Alzheimer's disease [10], and indicate that after traumatic brain injury (TBI) women show better recovery than men [9].

Apart from this clinical evidence, on which several gonadal steroids-oriented clinical trials have been based [11], relatively few studies have explored possible molecular mechanisms involved in gender-related differences. In this context, there has been much speculation concerning the gonadal hormone role in immune function and cytokines production during inflammation, myelination, and neurodegeneration/neuroprotection [4,5,12], but direct evidence regarding gender-related differences in these and other critical molecular and cellular hallmarks of the diseases is still very scanty.

We are exploring possible molecular determinants for gender-dependent differences in inflammatory demyelinating diseases using experimental allergic encephalomyelitis (EAE) in rats as a disease model for MS. EAE induced in Dark Agouti rats is Th1 mediated, having a relapsing-remitting course, and comprises also persistent demyelination, remyelination, neuronal distress and cognitive defects [13-18]. In this model, we have demonstrated by liquid chromatography-tandem mass spectrometry that the levels of neuroactive steroids display sex, regional and temporal differences in both control and EAE, and these changes did not correlate to the plasma levels of gonadal hormones [19,20].

In this paper data are presented regarding molecular markers for oligodendrocyte precursor cells, myelin proteins, neurotransmitters and neurotrophins in the course of EAE. Attention was focused on the spinal cord as a white matter-rich area in which most of the tissue is occupied by heavily myelinated tracts that is severely affected by inflammation and demyelination, and on the cerebellum as a grey matter-rich area since ataxia is a common symptom in EAE rats, and a cerebellar cortical atrophy has been described late in the disease in spite of poor inflammation and demyelination [21].

## Results

### Animals and disease progression

**Figure 1:**
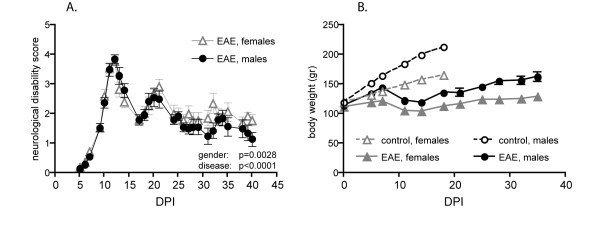
**Clinical score evolution (A) and body weight variations (B) in experimental animals (control female: white triangle, control male: white circle, EAE female: black triangle, EAE male: black circle)**. Significant differences were observed in the clinical disability score between male and female animals (two-way ANOVA, gender effect, **p = 0.0028, F(1,649) = 9.027; days post-immunization (DPI) effect, *** p < 0.0001, F(27,649) = 54.32.

The clinical profile of EAE is reported in Figure 1, where neurological disability score (A) and body weight graphs (B) are shown. Animals develop clinical signs for EAE starting from 7-8 days post-immunization (DPI), after which disease progressed rapidly until 14 DPI (acute phase). The first regression is not complete and at 21 DPI there is a relapse, which is not so severe as the acute phase. The recovery phase is followed until 40 DPI, when a very slight disability still persists. The statistical analysis by two-way ANOVA indicates that the course of the disease is different between male and female rats (p = 0.0028). The body weight curves show the physiological profile in male and female control rats, and the expected gain arrest in the acute phase, which is followed by a partial recovery (B).

**Figure 2:**
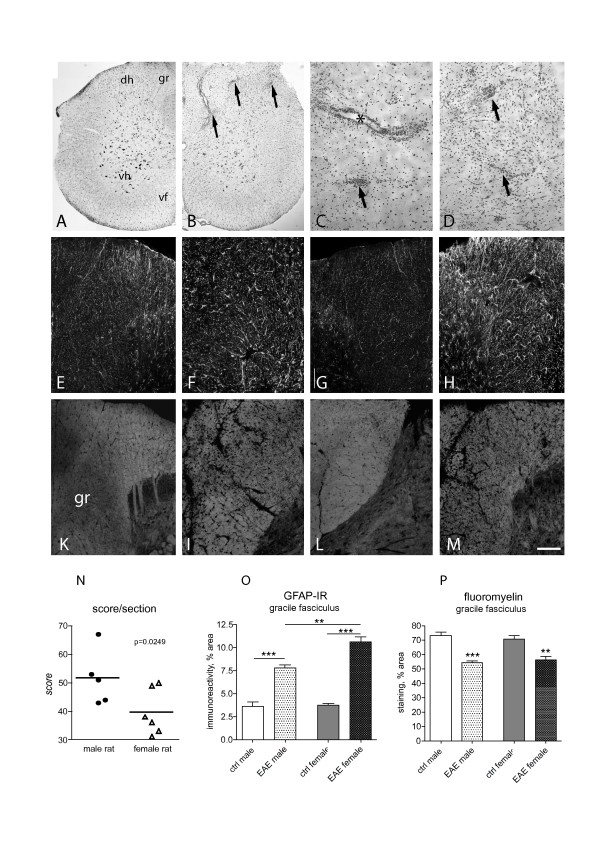
**Histopathology of the spinal cord in control and EAE animals was performed by E&E (A-D), GFAP-immunostaining for astroglial reaction (E-H) and FluoroMyelin staining for white tractd and myelin sheaths (K-M and related inserts)**. Micrograph in A illustrates a spinal cord hemi-sections in control, male rat; micrograph in B a spinal cord hemi-sections showing the extensive inflammatory infiltrates in EAE, as detailed in high power micrographs in C and D. Arrows indicate the intraparenchimal infiltrates asterisk in C a perivascular infiltrate. Image analysis indicates that there is a difference in the extension of inflammation infiltrate in female vs male EAE animals (N, Student's t test, p = 0.00249). Micrographs from E to H illustrate the astroglial reaction in EAE animals (F: female; H: male) with control animals (E: female; G: male). Image analysis indicates that astroglial reaction is stronger in female than in male EAE animals (O, one way ANOVA and Tukey multiple comparison test, **p < 0.01; ***p < 0.001). Micrographs from K to M illustrate white matter in EAE (I: female; M: male) compared with control animals (K: female; L: male). The color inserts refer to high power magnification, to shown the myelin sheath morphology. The original image has been processed using a deconvolution procedure. Image analysis indicates that demyelination is not different in female and male EAE animals (one way ANOVA and Tukey multiple comparison test, **p < 0.01; ***p < 0.001). Graph bars: M, 100 μm; color insert 10 μm.

Histopathology was analysed at 14 DPI. At this stage, a severe and diffuse inflammatory cellular infiltrate was observed in the spinal cord (Figure 2A: control; Figure 2B-D: EAE) in perivascular and intraparenchymal areas. The astroglial reaction was also analysed by GFAP-immunostaining (Figure 2E-H) and demyelination by FluoroMyelin histochemistry (Figure 2K-M). Morphometric analysis of the investigated markers is reported in Figure 2N-P. The semiquantitative evaluation of the inflammation score reveals a slight but significant difference between male and female rats, having the female animals a lower score (Figure 2N). The astroglial reaction observed in male and female rats during EAE was stronger in female than in male rats (Figure 2O). Demyelination, as analysed in the gracile fasciculus of the lumbar spinal cord, was no different in male and female rats.

At this stage, and as already reported in previously published paper [13] very few cellular infiltrates were found in cerebellum and cerebral cortex *(not shown)*.

### Myelin markers

**Figure 3:**
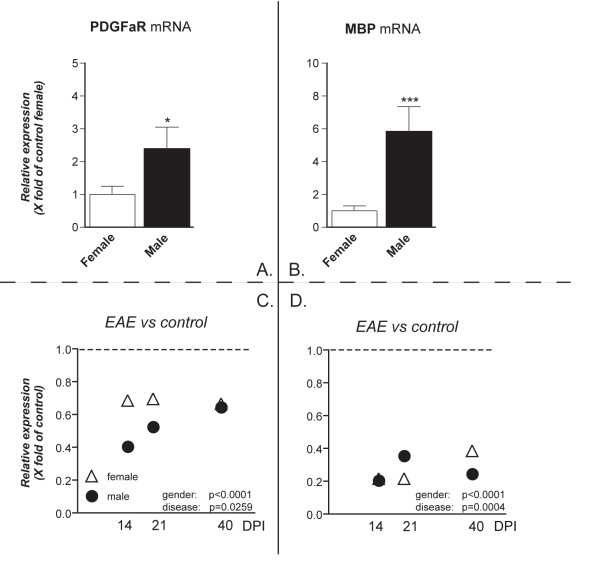
**PDGFαR and MBP mRNAs expression level in the spinal cord**. mRNA levels were studied by real-time PCR. Results are expressed as relative gene expression, from data obtained by using the equation 2^-ΔΔC^_T _. Results are presented as mean ± SEM. Eight animals were included in each group (male and female) at each time point. Panels A and B refers to the gender effect in control groups, panels C and D refers to the disease effect. PDGFαR mRNA expression level was higher in control males than in control females (A. Student's t test *p = 0.0175), and MBP mRNA in control males was 6 times higher than in control females (B. Student's t test ***p < 0.0004). C: Relative expression of PDGFαR mRNA temporal profile in EAE animals at 14, 21 and 40 DPI, in females (white triangle) and males (black circle), compared with respective/own sex control group. The statistical analysis (two-way ANOVA) indicates a gender effect (*** p < 0.0001, F(1,50) = 18.08) and a disease effect (* p = 0.0259, F(3,50) = 3.358; Bonferroni post-test: control, * p < 0.05). D: Relative expression of MBP mRNA in EAE at the different disease phases compared with control sex group. The statistical analysis (two-way ANOVA) indicates a gender effect (*** p < 0.0001, F(1,46) = 51.51) and a disease effect (*** p = 0.0004, F(3,46) = 7.454; Bonferroni post-test: control, ** p < 0.01; 14 DPI, ** p < 0.01; 21 DPI, *** p < 0.001; 40 DPI, * p < 0.05).

In order to investigate possible gender effects on demyelination/remyelination during EAE in Dark-Agouti rats, the expression level of myelin markers was analyzed in both male and female rats at the different phases of the EAE, in spinal cord and cerebellum. In particular, mRNA expression level was analyzed for PDGFαR, which is a marker for the oligodendrocyte precursor cells (OPCs) responsible for myelin repair in the mature CNS [22] and MBP, which is one of the most abundant among the myelin proteins [23]. In the spinal cord, PDGFαR mRNA expression in healthy males is around 2.5 times higher than in healthy females (p = 0.0175), whereas the MBP mRNA level is 5 times higher in males than in females (p = 0.0004) (Figure 3A, B). In both sexes, the expression of both genes was down-regulated in all phases of the disease (Figure 3C, D) and a significant difference was observed between females and males (two-way ANOVA and Bonferroni post-test). PDGFαR mRNA down-regulation is stronger in male than in female at 14 and 21 DPI. MBP mRNA expression level differs between male and female at 21 and 40 DPI. The histochemical analysis of myelin during the acute phase of EAE indicated that the decrease in myelin staining in the *gracile fasciculus *was comparable in both male and female rats (Figure 2C).

**Figure 4:**
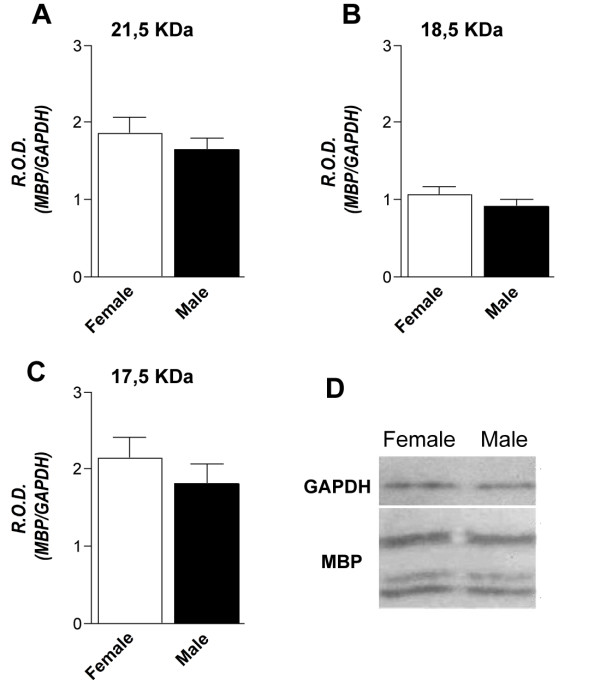
**Spinal cord MBP protein level in control rats**. The levels of MBP protein, isoforms 21.5, 18.5 and 17.5 KDa were studied using Western-blot. The graphs in A, B and C show the results of densitometric analysis after normalizing MBP with GAPDH (38 KDa). Results are expressed as mean R.O.D. (relative optical density) values ± SEM. D: representative blot with the bands corresponding to female and male GAPDH (38 KDa) and MBP 21.5 18.5 and 17.5 KDa isoforms. No differences were found between male and female control rats for the investigated MBP isoforms.

To determine whether the sex-dependent difference in MBP mRNA expression also had an impact on protein content, MBP protein levels were measured in the spinal cord of control healthy rats using Western blot. The 17.5, 18.5 and 21.5 isoforms were studied, and a representative Western-blot experiment has been included in Figure 4(D). No differences between female and male rats were observed in any of the investigated isoforms (Figure 4A, B, C). A morphometric analysis of the myelin sheath in male and female rats was also performed by measuring the G-ratio of the *lateral, dorsal *and *ventral funiculus *of the lumbar spinal cord in confocal images of FluoroMyelin staining. No differences between male and female rats were observed *(data not shown)*.

**Figure 5:**
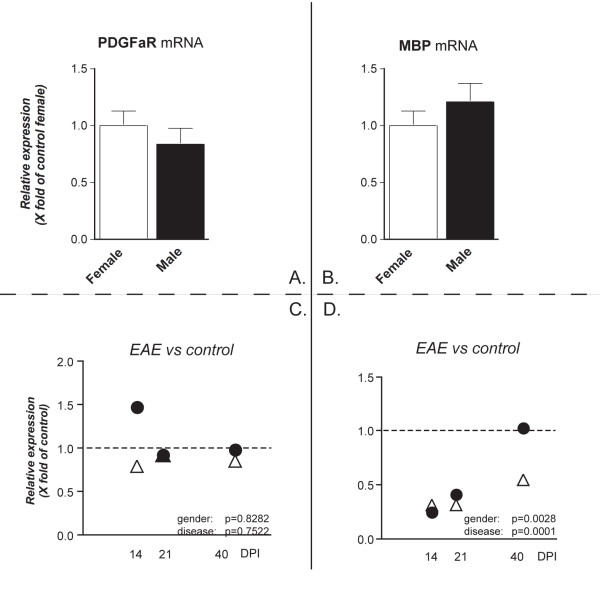
**PDGFαR and MBP mRNA expression level in the cerebellum**. mRNA levels were studied by real-time PCR. Results are expressed as relative gene expression, from data obtained by using the equation 2^-ΔΔC^_T _. Results are presented as mean ± SEM. Eight animals were included in each group (male and female) at each time point. Panels A and B refers to the gender effect in control groups, panels C and D refers to the disease effect. No differences were observed in PDGFαR and MBP mRNA expression in female and male healthy controls (A, B), such as in PDGFαR mRNA expression in female (white triangle) and male (black circle) EAE animals at different DPI (C). On the contrary, statistical analysis (two-way ANOVA) shows that relative MBP mRNA expression in EAE animals in different in male and female rats (** p = 0.0028, F(1,52) = 9.831) and at different time points (*** p < 0.0001, F(3,52) = 25.14; Bonferroni post-test: 40 DPI, ** p < 0.01).

In the cerebellum, no significant changes were observed in the expression of PDGFαR mRNA between healthy male and female rats and during the course of EAE (Figure 5A, C). On the contrary, MBP mRNA expression level was down-regulated during EAE in both females and males. Significant differences were observed in the expression of the MBP gene in EAE animals (Figure 5B, D) at 40 DPI between females and males, being lower in female than male rats (Bonferroni test, ** p < 0.01).

### GAD, neurotrophins and related receptors

**Figure 6:**
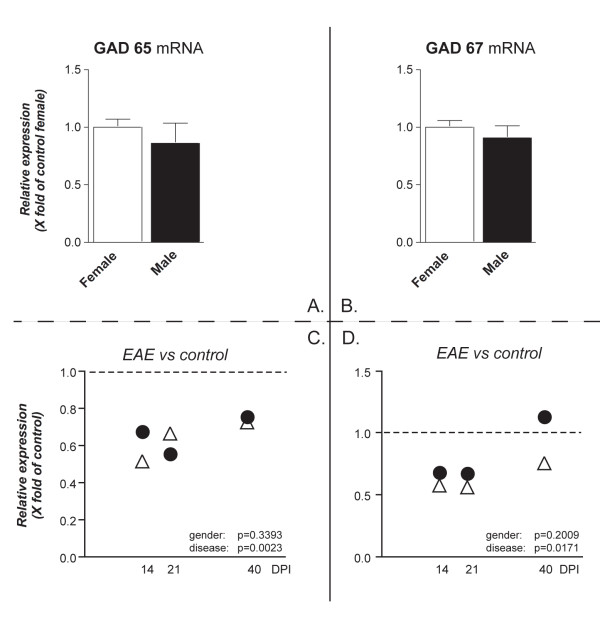
**GAD65 and GAD67 mRNAs expression level in the cerebellum**. mRNA levels were studied by real-time PCR. Results are expressed as relative gene expression, from data obtained by using the equation 2^-ΔΔC^_T _. Results are presented as mean ± SEM. Eight animals were included in each group (male and female) at each time point. Panels A and B refers to the gender effect in control groups, panels C and D refers to the disease effect. No differences were observed in GAD65 and GAD67 mRNA expression in female and male healthy controls (A, B). Statistical analysis (two-way ANOVA) shows that relative GAD65 and GAD67 mRNA expression in EAE animals is similar in male (white triangle) and female (black circle) rats (p = 0.3393 and p = 0.2009, respectively), whereas both mRNAs differ at different time points (disease effect: GAD65 **p = 0.0023, F(3,52) = 5.528; GAD67 *p = 0.0171, F(3,52) = 3.708).

In order to investigate markers for neuronal distress/lesion, the expression level for mRNAs encoding for GABA synthesis enzyme and neurotransmitter and neurotrophins was analyzed. No gender effect was observed in the expression of mRNA encoding for the enzymes GAD65 and GAD67 either in healthy or EAE animals (Figure 6A, B). However, the expression of GAD65 and GAD67 mRNA during EAE was down-regulated in both male and female rats. Significant differences were observed at different phases of EAE. GAD65 and GAD 67 mRNA levels displayed a recovery at 40 DPI.

**Figure 7:**
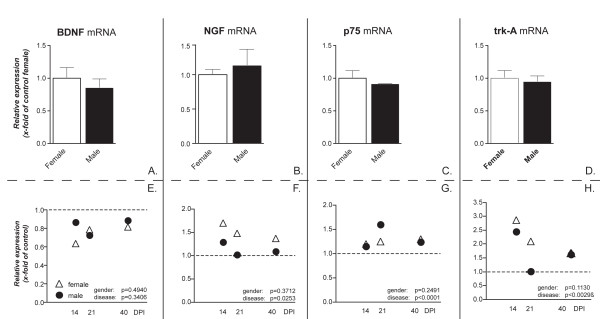
**NGF, BDNF, p75 and trkA mRNAs expression level in the cerebellum**. mRNA levels were studied by real-time PCR. Results are expressed as relative gene expression, from data obtained by using the equation 2^-ΔΔC^_T _. Results are presented as mean ± SEM. Eight animals were included in each group (male and female) at each time point. Panels A to D refers to the gender effect in control groups, panels E and H refer to the disease effect. No differences female and male healthy controls were observed in this group of transcripts (A, B). Statistical analysis (two-way ANOVA) shows that mRNA expression in EAE animals is similar in male (black circle) and female (white triangle) rats for all transcripts, whereas they differ at different time points (NGF: *p = 0.0253, F(3,53) = 3.365; p75: *** p < 0.0001 F(3,52) = 9.878; trkA: ** p = 0.0029, F(3,52) = 5.299).

The expression of BDNF, NGF neurotrophin receptors trkA (high-affinity NGF receptor) and p75 (low affinity NGF and BDNF receptor) was also studied during EAE in both sexes in the cerebellum. No gender effect for any of the investigated mRNAs was found in healthy animals (Figure 7A, B, C, D). BDNF mRNA expression was not significantly changed during the course of EAE (Figure 7E) in both male and female rats. On the contrary, there was a significant up-regulation of NGF mRNA expression level in the acute, inflammatory phase of the disease in both male and female rats (Figure 7F, 14 DPI), which was partly attenuated, but not resolved, in the course of the disease in female rats and resolved already at 21 days in male rats. A slight, but significant and stable up-regulation of the NGF low-affinity receptor p75 mRNA expression was observed in both female and male rats (Figure 7G). An up-regulation of the NGF high-affinity receptor trkA mRNA expression level was also found in female and male (Figure 7H).

## Discussion

Gender bias in autoimmune diseases is a well-known and hitherto unexplained fact. In particular, MS is more prevalent in females than in males, and this female predominance increases as time goes by. Gender appears to play a critical role also in the progression of MS, suggesting that not only immune reaction, but also remyelination, axonal pathology and neural damage might be gender-dependent [24]. In all cases, the histopathological and molecular mechanisms underlying the inherent differences in male and female MS are still obscure [25].

In the present study molecular markers for myelin and grey matter that are differentially regulated in male and female rats in the experimental model of the disease have been described. Two areas were investigated: the spinal cord, which is the area with extensive inflammation and demyelination, and the cerebellum, in view of the ataxia symptom in EAE, where inflammatory cellular infiltrates are scare in this disease model [26]. While the clinical profile of the disease differs between sexes being more severe in female than male rats, the inflammatory cellular infiltration in the spinal cord is lower in females, but produces a stronger astroglial reaction than in males. Moreover, disease-induced alteration of several markers is different between the two sexes. In particular, a strong gender-dependent difference in MBP and PDGFaR mRNA expression level in the spinal cord of healthy animals was found, which correspond to a different regulation during the disease.

### EAE in male and female Dark Agouti rats

Here we confirm that both male and female Dark Agouti rats are highly susceptible to EAE [26]. While the clinical score profile in the acute phase is similar in males and females, male rats show a more pronounced recovery than female rats. In this EAE model, a massive infiltration by inflammatory cells, and a massive demyelination were observed in the spinal cord, whereas small and localized lesions were spread over the main white tracts, including the cerebellar peduncoli, corpus callosum and optic nerve [15,16,26]. The remyelination process starts quite early also in Dark Agouti rats, but it is not yet complete at 40 DPI [16]. In spite of the fact that severity of inflammatory infiltrate in the spinal cord is lower in female compared to male rats, astroglial reaction is more pronounced in female than in male rats. This could be related to humoral rather cellular immune reaction. Overall, the females of all the species used for MS models display stronger immune responses than males [26-28]. This is attributed to cytokines production [29,30]. For example, cytokine IL-13 is implicated in gender differences in EAE severity in C57BL/6 mice, where the absence of the anti-inflammatory IL-13 entails lower susceptibility to EAE in females vs males or WT females with normal levels of IL-13 [31].

### Gender-dependent differences in white matter during health and inflammatory-demyelinating disease

When comparing the expression level of the genes included in the study in male vs female healthy rats, the most significant result was the 5-fold higher MBP and the 2.5-fold higher PDGFαR expression in males than females in the spinal cord, but not in the cerebellum. This difference is not present at protein level, and, more generally, no significant differences in myelin sheaths in the spinal cord were found between male and female rats. This result might suggest that MBP synthesis could be regulated in a different way at post-transcriptional and translational levels in males and females. In fact, mRNA levels and protein levels do not always correlate [32,33] and this might be due to the post-transcriptional mechanisms playing and/or to the different in vivo half life of proteins. Gonadal steroid and neurosteroid may elicit effects through non-genomic mechanisms via ERs localized on the plasma membrane, and ligand-independent pathways to activate ERs have been also described [34]. Moreover, the mechanisms controlling the rates of degradation/synthesis for a given mRNA and protein are not homogeneous, even within proteins that have similar functions [33]. Technical limits of the methods for quantifying mRNA and proteins should also be taken into account.

More generally, the issue of gender-related difference in the anatomy of white tracts is complex and controversial. The gender-dependent difference in PDGFαR and MBP mRNA in the spinal cord correlates with data from Cerghet *et al*. [35,36] in mice. They found that the density of oligodendrocytes and the content of several myelin proteins in white tracts is higher in males than in females, whereas the lifespan of oligodendrocytes is shorter in females than in males, thus suggesting that myelin turnover is greater in females than in males. It may thus be argued that males have a greater functional reserve than females, whereas females have a greater vulnerability related to higher myelin protein turnover.

We and others already described the variation of MBP protein content, such as different markers for OPCs in male and female rats during EAE [13,15,16,37]. In this study we report that there is a gender-dependent difference in the regulation of PDGFαR and MBP during the experimental disease, involving both genes in the spinal cord, and MBP, only in the cerebellum. This correlates with other reports describing differences in remyelination in old male and female rats [38] and in cuprizone intoxication model (reversible inflammatory demyelination) [39].

### Neurotrophins and neuronal markers in inflammatory-demyelinating diseases

The EAE model in rodents (and primates) allows neuronal distress/damage to be investigated. We already described how the expression level of the acetylcholine synthesis enzyme choline acetyltransferase mRNA level was transiently reduced in motor neurons in the spinal cord [17] and in cholinergic neuron of the basal forebrain [18] during EAE. Here investigation was focused on cerebellum, which is involved in motor symptoms in EAE. In spite of scant inflammation and demyelination, a grey matter atrophy [21] and a Purkinje cell loss [40] has been described in the cerebellum. As index of neuronal injury, we investigated the expression level of the mRNAs encoding for the GABA synthesis enzyme γ-aminobutyric acid decarboxylase (GAD) [41]. A transient down-regulation of both GAD65 and GAD67 was observed in male and female EAE rats, thus confirming that the acute phase of the disease is associated with reversible neuron distress.

Due to the neurotrophins role as endogenous neuroprotectors, their expression levels were investigated during EAE, focusing on NGF and BDNF. Previous results from our laboratory have described higher levels of NGF in certain brain areas, like the thalamus and cerebral cortex, but not the spinal cord, in EAE compared to healthy rats, associated to a strong up-regulation of p75- and trkA-like immunoreactivity [13]. This up-regulation diminishes over time and a drop in NGF mRNA expression level was reported in the cerebral cortex at 104 DPI [18]. Here we confirm the up-regulation of NGF and its high-affinity receptor trkA mRNA in the cerebellum in both male and female in the early, inflammatory phase of EAE, while BDNF is down-regulated at the same times. NGF, but also BDNF, modulate inflammation and immune cell function in many diseases [42-45]. Both the high and low affinity NGF receptors are widely expressed in the immune system, thus indicating a potential for responding to this neurotrophin through an autocrine mechanism [43,45]. NGF increase during EAE may possibly also result in increased neuroprotection [46], working with the marmoset model, showed that NGF icv administration delayed the onset of clinical EAE, and also prevented the full development of EAE lesions. NGF administration also influences EAE development and progression in rats [47] by reducing the severity of the disease compared to that in saline treated EAE mice.

The low-affinity receptor p75 is also up-regulated. Notably, the up-regulation of p75 in Purkinje neurons has been described in EAE [48]. The p75 up-regulation in EAE could be related to the Purkinje neurons death and cerebellar atrophy, since p75 can induce autophagy and death in these cells [49].

Since the original report describing the different content of NGF in the submaxillary gland in male and female mice [50], many other reports have illustrated sexually dimorphic distribution of NGF in tissues and plasma of different animal species [51-53]. The cerebellum and particularly the Purkinje cells have been recognized as a major source for neurosteroid production [54], and we reported that neurosteroids are differentially regulated in the cerebellum of control male and female rats, such as during EAE [20]. Our mRNA data indicate that there are no sex differences in the NGF and BDNF levels in cerebellum either in healthy and EAE rats, thus confirming previous reports suggesting that neurotrophin levels do not correlate with estrogen levels in females or with estrogen or testosterone levels in males at this age [55,56].

## Conclusions

Differences in vulnerability and disease evolution have already given rise to clinical trials for MS based on gonadal steroids. With regard to animal models of inflammatory-demyelinating diseases, it has recently been reported that the combined administration of 17 beta-estradiol and progesterone prevents cuprizone-provoked demyelination of the corpus callosum in male mice [57]. A similar effect of combined treatment was also found in MOG (40-45)-induced EAE mice [58]. However, rational future prospects regarding the use of sex steroids as adjuvant therapy in MS should be based on the identification of the pathological process, cell type, and molecular pathways positively affected by steroids with regard to disease onset and progression. This study provides a description of the gender-dependent and disease-dependent regulation of markers for these different processes in a well-established animal model, suggesting that demyelination/remyelination might be a target for gender-dependent therapies.

## Methods

### Animals, EAE induction, groups size

Dark-Agouti (Harlan, Italy) male and female rats, 150-175 g body weight were used in this study. In both female and male rats, a group was sensitized with a medium containing 0.15 g/ml guinea pig spinal cord tissue in complete Freund's adjuvant (CFA, Sigma), 50% v/v, to which 5 mg/ml of heat-inactivated Mycobacterium (Difco H37Ra) was added. Un-injected rats were used as controls. Rats were regularly weighed and examined for clinical signs of EAE by a trained observer according to a semiquantitative score: 1 = loss of tail tone, 2 = mono or bilateral weakness of hind legs or middle ataxia, 3 = ataxia or paralysis, 4 = severe hind legs paralysis, 5 = severe hind leg paralysis and urinary incontinence. To determine the different phases of the estrous cycle, female rats were monitored by daily vaginal smears and only those demonstrating at least two consecutive 4-day cycles were used in the study and killed on the day of proestrus. Tissues were collected in two independent experiments, the first for molecular biology studies, the second for morphology and western blotting. Sixty-four animals (32 females and 32 males) were included in the first experiment; eight animals were included in each time-point groups (sacrificed 14, 21, 40 DPI) for both male and female, and for un-injected animals. Twenty-four animals (12 females and 12 males) were include in the second experiment, were tissue was collected at 14 DPI. All animal protocols described herein were carried out according to the European Community Council Directives (86/609/EEC) and approved by the intramural ethical committee for animal experimentation of Bologna University and Ministry of Health, comply with the guidelines published in the *NIH Guide for the Care and Use of Laboratory Animals*.

### Histology, histochemistry and immunohistochemistry

Six animals/group were included in this part of the study. EAE animals were killed at 14 DPI, i.e. during the acute phase of the disease. The lumbar tract of the spinal cord was rapidly dissected out, briefly washed in PBS and then fixed using a paraformaldehyde 4% + saturated picric acid 14% solution in PBS 0.2 M, pH 6.9 for 12 hours (h). Cryostat sections (14 μm) were then collected from the spinal cord. The extent of inflammation, demyelination and astroglial rection was evaluated on tissue sections stained with hematoxylin and eosin to visualize infiltrate cells, with FluoroMyelin™ Fluorescence Myelin Staining (Molecular Probes, Eugene, OR) for the myelin sheaths. Indirect immunofluorescence (IF) procedures were used to visualize the anti-fibrillary acid protein (GFAP, Chemicon International Inc. Temecula, CA, USA). Briefly, sections were first incubated in 0.1 M phosphate buffered saline (PBS) at room temperature for 10-30 min, followed by incubation at 4°C for 24 h in a humid atmosphere with the primary antibodies diluted in PBS containing 0.3% Triton X-100, v/v. After rinsing in PBS for 20 min (2 × 10 min), sections were incubated at 37°C for 30 min in a humid atmosphere with the secondary antisera conjugated with different fluorochromes diluted in PBS/Triton X-100 0.3%. Sections were then rinsed in PBS (as above) and mounted in glycerol containing 1,4-phenylendiamine 0.1 g/l (Sigma). Images were taken by Olympus AX70-PROVIS microscope equipped with motorized z-stage control and F-VIEW II CCD Camera. The inflammatory infiltration was evaluated by two independent operators in blind on 5 replicate sections per animal, by counting the number and severity of cellular infiltrates over each, entire coronal section. Cellular infiltrates were scored as follows: 0, none; 1, a few inflammatory cells; 2, organization of perivascular infiltrates; 3, increasing severity of perivascular cuffing with extension into the adjacent tissue [59-61]. The inflammation score, expressed as score/section, derives from the sum of infiltration score in each cellular infiltrate. To minimize the bias due to random distribution of the demyelinating lesions, the FluoroMyelin and GFAP-staining were both measured in the fasciculus gracilis, and calculated as percentage positive areas using the Image ProPlus software (Imaging Research Inc, St. Catharines, Ont., Canada). Myelin sheath thickness was measured on confocal images (Olympus FluoView 500) using Image ProPlus software (MediaCybernetics, Bethesda, MD). The G-ratio (ratio of axon diameter to total fibre diameter) was calculated on confocal images by dividing the circumference of an axon without myelin by the circumference of the same axon including myelin. At least 250 fibers/group were included in the analysis.

### RNA isolation, reverse transcription and Semiquantitative real-time PCR

Total RNA was prepared by following manufacture's instructions (Mini RNeasy Kit, Qiagen, Milan, Italy). RNAs were first subjected to DNase treatment (0.1 U/μl, 1× DNase buffer, 4 U/μl RNase inhibitor, all from Fermentas, Life Sciences, Milan, Italy) by incubating at 37°C for 30 min. First strand cDNAs were obtained using 10 U/μl of the M-Moloney murine leukaemia virus (MuLV) reverse transcriptase enzyme (Fermentas), 1 mM of each d(NTP)s (Fermentas), 5 μM of pd(N)_6 _random primers (Roche, Molecular Biochemical's, Indianapolis, IN, USA) and 1 U/μl oligo(dT)_18 _(Fermentas). In order to discard possible contamination of genomic DNA in isolated RNAs, one sample with no reverse transcriptase enzyme (no RT sample) was processed in parallel to the others and tested by real-time PCR for every pair of primers used. PCR reactions were performed with the Mx3005*P*™ real-time PCR system (Stratagene, CA, USA) using SYBR-green I dye. All primers used for SYBR-green real-time PCR were designer with the Beacon Designer software (BD 5.0, Premier Biosoft International, Palo Alto, CA, USA); primer sequences have been included in Table 1. The housekeeping gene GAPDH was used to normalize the amount of retro-transcribed mRNA used for PCR. Reactions were performed in a final volume of 25 μl consisting on 1× master mix, (Fermentas) and 0.4 μM of both forward and reverse primers. Two steps were performed: 1. denaturation (95°C, 10 min); 2. annealing/extension (40 cycles of: 95°C for 15 sec, 60°C for 30 sec); at the end of PCR reaction the melting curve of amplified products was always performed.

**Table 1:** Nucleotide sequences of primers used for gene expression study by real-time semiquantitative PCR assays.

Gene	Primer	Sequence
BDNF	Forward reverse	5'-gtgacagtattagcgagtg-3' 5'-gccttccttcgtgtaacc-3'
GAD65	Forward reverse	5'-gcccgctataagatgtctc-3' 5'-aatcacactgtctgttcc-3'
GAD67	Forward Reverse	5'-ggcatcttccactccttc-3' 5'-gacgactcttctcttccag-3'
MBP	Forward reverse	5'-catccttgactccatcgg-3' 5'-tttcatcttgggtcctctg-3'
NGF	Forward reverse	5'-gacgactcttctcttccag-3' 5'-cgtggctgtggtcttatctc-3'
PDGFαR	Forward reverse	5'- ctggtgcctgcctcctac-3' 5'- aactcgctggtcttgaacg-3'
p75	Forward reverse	5'-agtggcatctctgtggac-3' 5'-ctacctcctcacgcttgg-3'
trk-A	Forward reverse	5'-tgaatctgtcctccaatgc-3' 5'-tgaagcgtctgtgtatgc-3'
GAPDH	Forward reverse	5'-ggcaagttcaatggcacagtcaag-3' 5'-acatactcagcaccagcatcacc-3'

The efficiency of each pair of used primers was calculated by amplifying cDNA serial dilutions in the conditions above described obtaining efficiency values in the range of 95-102%. The 2^^(-ΔΔ*C*^_T_^) ^method was used for the calculation of gene expression relative to a given reference group (*C*_T_, threshold cycle). When comparing gene expression between sexes,ΔΔ*C*_T _was calculated as follow: ΔΔ*C*_T _= (*C*_T target gene_- *C*_T GAPDH_) female - (*C*_T target gene_- *C*_T GAPDH_) male. When instead comparing gene expression between healthy (Control) and EAE animals at different phases of the disease, the ΔΔ*C*_T _formula was: ΔΔ*C*_T _= (*C*_T target gene_- *C*_T GAPDH_) Control - (*C*_T target gene_- *C*_T GAPDH_) EAE acute (14 DPI), ΔΔ*C*_T _= (*C*_T target gene_- *C*_T GAPDH_) Control - (*C*_T target gene_- *C*_T GAPDH_) EAE remission (21 DPI), ΔΔ*C*_T _= (*C*_T target gene_- *C*_T GAPDH_) Control - (*C*_T target gene_- *C*_T GAPDH_) EAE chronic (40 DPI).

The specificity of real-time PCR reactions was evidenced by the melting curve of the amplified products, obtaining a unique peak at the correspondent melting temperature (Tm) (Figure 3E). No C_T _was obtained after real-time PCR of no RT sample for any of the pair of primers used. Template controls gave always no *C*_T _as well. Random amplified products were resolved in a 2.5% agarose gel and TAE electrophoresis buffer, obtaining a unique band of the expected size. DNA marker of 100 bp ladder (Fermentas) was used.

### Western blotting procedure

Tissue homogenates were prepared using a lysing buffer consisting on 10 mM Hepes, 1 mM DTT, pH 7.5 and protease inhibitor cocktail (Sigma). Equal amounts of protein, concentrations determined by Lowry method with the Protein assay kit (Bio-Rad, Hercules, California, USA), were separated in 15% SDS-polyacrylamide gels and electroblotted to nitrocellulose membranes. In order to block nonspecific protein binding sites, filters were incubated with blocking solution (Pierce, Rockford, IL, USA) for 2 h at room temperature and primary antibodies were then incubated overnight at 4°C. After washing for 1 hour with TTBS (TBS-0.05% Tween-20), filters were incubated with secondary antibodies for 30 min at room temperature and washed again for another hour. Rabbit polyclonal anti-MBP (DAKO), dilution 1:2000 and GAPDH (glyceraldehyde 3-phosphate dehydrogenase) (Millipore, Milan, Italy), dilution 1:150 were used as primary antibodies while anti-rabbit (1:30,000) and anti-mouse (1:2000), respectively, antiserum conjugated to horseradish peroxidase (DAKO) were used as secondary antibody. Finally, proteins were detected using an ECL chemioluminescent kit (Pierce) and exposition to radiographic film. Densitometric analysis was performed using the AIS Imaging System software (Ontario, Canada) and the data obtained statistically analysed and represented using PrismGraph software (GraphPad Software, San Diego, CA, USA). Seven animals/group were used to perform these studies.

### Data presentation and statistical analysis

For mRNAs expression data, we first analyzed the difference between female and male control animals (data normalized vs female, because the disease is usually induced in female rats.). Student's t test was used for the statistical analysis. Since the aim of the study was to investigate the disease effect at different time point in male and female rats, we then analyzed the disease and gender influence by two-way ANOVA and Bonferroni post-test. Results for two-way ANOVA are reported in the figures and figure legends. Morphological data were analyzed by Student's t test, and clinical data by two-way ANOVA. Results were considered significant when the probability of their occurrence by chance alone was less than 5%. GraphPad Software (San Diego, CA, USA) was used for statistics and graph preparation.

## Abbreviations

BDNF: Brain-derived neurotrophic factor; CFA: complete Freund's adjuvant; CNS: Central nervous system; DPI: day post-immunization; EAE: experimental allergic encephalomyelitis; GAD: γ-aminobutyric acid decarboxylase; GABA: γ-aminobutyric acid; GAPDH: glyceraldehyde 3-phosphate dehydrogenase; GFAP: anti-fibrillary acid protein; MBP: myelin basic protein; MOG: myelin oligodendrocyte glycoprotein; MS: Multiple sclerosis; NGF: Nerve growth factor; OPCs: oligodendrocyte precursor cells; PDGF: platelet-derived growth factor.

## Competing interests

The authors declare that they have no competing interests.

## Authors' contributions

AM: carried out the molecular biology studies. GD: carried out the EAE induction, the histology, histochemistry, immunohistochemistry and morphometry. MF: carried out molecular biology studies, Western blotting procedure and statistical analysis. SS: carried out the histology, histochemistry and immunohistochemistry and morphometry.

LL: carried out the EAE induction and followed animals clinically. SG: carried out Western blotting procedure. RCM: participated in the design of the study and drafted the manuscript. LC: conceived of the study, participated in its design and coordination and redacted the manuscript. LG: participated in the design of the study and redacted the manuscript. All authors read and approved the final manuscript.
